# DXA-derived biofidelic finite element models for quantifying the efficacy of hip protectors

**DOI:** 10.1093/jbmrpl/ziag067

**Published:** 2026-04-14

**Authors:** Dheeraj Jha, Yijun Zhou, Anitha D Praveen, Ellie S Galliker, Preeti Gupta, Ecosse L Lamoureux, Namki Hong, Yumie Rhee, Halldór Pálsson, Stephen J Ferguson, Benedikt Helgason

**Affiliations:** Future Health Technologies, Singapore-ETH Centre, CREATE Campus, Singapore 138602, Singapore; Institute for Biomechanics, Department of Health Sciences and Technology, ETH Zürich, 8092 Zürich, Switzerland; Future Health Technologies, Singapore-ETH Centre, CREATE Campus, Singapore 138602, Singapore; Future Health Technologies, Singapore-ETH Centre, CREATE Campus, Singapore 138602, Singapore; Institute for Biomechanics, Department of Health Sciences and Technology, ETH Zürich, 8092 Zürich, Switzerland; Population Health Research Platform, Singapore Eye Research Institute (SERI), Singapore 169856, Singapore; Department of Health Services & Systems Research, Singapore, Duke-NUS Medical School, Singapore 169857, Singapore; Singapore National Eye Centre (SNEC), Singapore 168751, Singapore; Population Health Research Platform, Singapore Eye Research Institute (SERI), Singapore 169856, Singapore; Department of Health Services & Systems Research, Singapore, Duke-NUS Medical School, Singapore 169857, Singapore; Department of Internal Medicine, Research Institute of Endocrinology, Yonsei University College of Medicine, Seoul 06916, South Korea; Department of Internal Medicine, Research Institute of Endocrinology, Yonsei University College of Medicine, Seoul 06916, South Korea; Department of Industrial Engineering, Mechanical Engineering and Computer Science, University of Iceland, 102 Reykjavik, Iceland; Future Health Technologies, Singapore-ETH Centre, CREATE Campus, Singapore 138602, Singapore; Institute for Biomechanics, Department of Health Sciences and Technology, ETH Zürich, 8092 Zürich, Switzerland; Future Health Technologies, Singapore-ETH Centre, CREATE Campus, Singapore 138602, Singapore; Institute for Biomechanics, Department of Health Sciences and Technology, ETH Zürich, 8092 Zürich, Switzerland

**Keywords:** hip fracture probability, DXA, finite element modeling, hip protector, relative risk

## Abstract

We aimed to develop a tool to predict the probability of hip fracture given a fall, using biofidelic finite element models (FEMs) built from DXA scans. Additionally, we evaluated the effectiveness of hip protectors, assuming full compliance, by quantifying the reduction in relative risk in-silico. This study included 700 subjects (325 males and 375 females) from the Population Health and Eye Disease Profile in Elderly Singaporeans (PIONEER) cohort, consisting of Chinese, Indian, and Malay ethnicities. The probability of hip fracture was calculated using DXA-based biofidelic FEMs by simulating various falls. The relative risk was determined by comparing fracture probabilities between falls with and without hip protectors. The overall probability of hip fracture in the cohort (mean age: 71.5 yr) was 4.49% (SD: 4.71). Chinese males had a significantly higher fracture risk, with odds ratios of 3.15 (95% CI: 1.81-5.49) compared to Malay males, and 4.57 (95% CI, 2.21-9.44) compared to Indian males. Similarly, Chinese females had a higher fracture risk, with odds ratios of 2.22 (95% CI, 1.16-4.24) compared to Malay females, and 7.70 (95% CI, 2.30-25.76) compared to Indian females. The relative risk with hip protectors was 0.38 (SD: 0.28) for males overall, with subgroup values of 0.42 (SD: 0.29) for Chinese, 0.33 (SD: 0.29) for Indians, and 0.38 (SD: 0.26) for Malays. For females, the overall relative risk was 0.43 (SD: 0.28), with subgroup values of 0.41 (SD: 0.21) for Chinese, 0.37 (SD: 0.32) for Indian, and 0.51 (SD: 0.29) for Malay, with no significant differences between ethnicities. In conclusion, we have developed an in-silico tool to assess hip fracture risk and evaluate the effectiveness of hip protectors using clinical DXA scans. These findings support the adoption of hip protectors as a preventive measure to reduce fracture risk.

## Introduction

Hip fractures are a biomechanical event influenced by a complex combination of risk factors that lead to either weakened bone strength or an excessive impact load.^1^ Currently, hip fracture risk is assessed using T-scores derived from areal BMD (aBMD) measurements via DXA scans, and the fracture risk assessment tool-hip fracture probability (FRAX-HFP) score (https://frax.shef.ac.uk/FRAX/). However, T-score-based assessments have only moderate sensitivity,^2^ and recent findings indicate that the FRAX-HFP’s accuracy can vary by ethnicity.^3^^,^^4^ Recent studies have shown that femoral strength, derived from finite element models (FEMs), has superior discriminatory power compared to aBMD,^5–9^ but there is still a ceiling effect, leaving up to 20% of risk unaccounted for.^7^ This limitation arises because these models often classify subjects with weak bones as high fracture risk while overlooking the protective role of soft tissue, which could reduce fracture risk, and fail to account for fall risk. Impact load, a key factor in determining fall outcomes, refers to the force the subject experiences in the event of a fall, which depends on variables like fall angles and impact velocity. Biofidelic FEMs created from simulated DXA images of CT scans have been developed to predict fall outcomes with accuracy comparable to that of CT-derived FEMs.^10^ This technology has since transformed into an automated system for creating biofidelic FEMs directly from clinical DXA scans.^11^ These advanced fall simulation models, which incorporate soft tissue and the pelvis, hold promise with respect to being more sensitive in terms of identifying fracture risk than biomarkers of less fidelity, such as aBMD. This is related to soft tissue playing a protective role against hip fractures during falls.^12^ However, a primary challenge lies in predicting the probability of a hip fracture in the event of a future fall, the configuration and circumstances of which are hard to predict.

Once an elevated risk is established, mitigation in current clinical practice mostly relies on bone-enhancing agents, though alternative approaches, such as fall-prevention exercise programs^13^ and hip protectors, are also available. Hip protectors are pads worn on each side of the hip that help reduce the impact force on the hip during a fall, offering immediate protection.^14^ However, systematic reviews on the efficacy of hip protectors have yielded inconsistent results,^15^^,^^16^ likely due to variations in study inclusion criteria and the specific types of hip protectors examined.^17^ A recent study showed that hip protectors can reduce the relative risk by using attenuation coefficients in an in-silico clinical trial with virtual patients.^18^ However, this low-fidelity model does not consider subject-specific energy transfer,^19^ as it does not model the entire hip or simulate the fall dynamics. Fleps and colleagues found that more than half of the total energy remains in the form of kinetic energy at the time of peak impact force, since the tissues around the pelvis are still moving and the pelvis continues to rotate.^19^ In a prior study, Galliker and colleagues evaluated the effectiveness of a generic hip protector design using biofidelic FEMs and found it could enhance force attenuation during lateral and posterior-lateral falls.^20^ However, the effectiveness of this generic hip protector in preventing hip fractures in a broader population, under broader fall conditions, has not yet been investigated.

Therefore, the primary aim of this study was to predict the probability of hip fracture given a fall (P_Fx_), using biofidelic FEMs built from DXA images acquired for a cohort of community-dwelling older adults in Singapore. A secondary aim was to use this technology to quantify the relative risk (RR) by assuming full compliance of hip protectors.

## Materials and methods

### Study population

This study included 700 older adults from the Population Health and Eye Disease Profile in Elderly Singaporeans (PIONEER) cohort,^21^ which consists of a random sample of 2643 community-dwelling individuals aged 60 yr and above from Singapore. The cohort represents the three major ethnic groups in Singapore: Chinese, Indian, and Malay. Data collection occurred between 2017 and 2022, with ethical approval granted by the SingHealth Centralized Institutional Review Board (approval number 2016/3089). Demographic information such as age, height, weight, and BMI was recorded for each participant. Eligibility criteria for inclusion in the study required that subjects had available data from FRAX-HFP assessments, DXA scans, and DXA-based FEM analysis. To determine the sample size required to assess the effectiveness of hip protectors, we calculated an effect size (Cohen’s *d*) of 0.81 based on total force measurements recorded with and without the hip protector.^14^ With a significance level (*α*) of 0.05 and a statistical power of 0.90, it was determined that a minimum of 32 subjects per group was necessary to detect a significant difference. To ensure adequate representation of both sex and the three main ethnic groups, the final sample size was set at 210, with 35 subjects per ethnicity for each sex.

### aBMD measurements and FRAX-HFP calculations

Subjects underwent DXA scans of the hip, and whole-body, performed using the Hologic Horizon W scanner. The aBMD and T-score at the femoral neck (FN) were extracted from the DXA scan reports generated by the scanner software. The FRAX-HFP score, which estimates the 10-yr probability of a hip fracture, was calculated using 12 clinical risk factors available in the FRAX tool (https://frax.shef.ac.uk/FRAX/). These risk factors include age, sex, weight, height, prior fracture history, parental history of hip fracture, current smoking status, glucocorticoid use, RA, secondary osteoporosis, alcohol consumption, and aBMD at the FN.

### DXA-based biofidelic FEM

**Figure 1:**
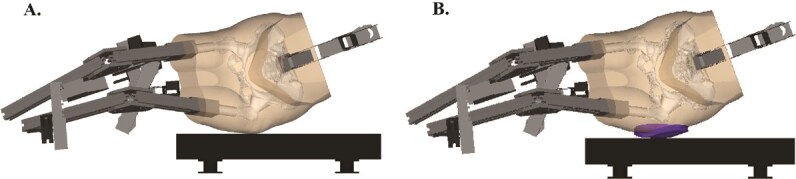
(A) DXA-based biofidelic finite element model representing a sideways fall, generated by converting a 2D DXA scan into a 3D femoral geometry with density-based material properties and subject-specific soft tissue estimates.^11^ (B) Same model with a hip protector.

An automated pipeline for building DXA-based biofidelic FEMs of the hip for simulating the effect of falls on the skeletal structures was developed in a previous study.^11^ The pipeline is briefly described here for clarity and context. The commercial software 3D-SHAPER was employed to transform the 2D hip DXA scan into a 3D volume along with associated density values.^22^ Further, a post-processing workflow was applied to the resulting 3D volume and density to align strength predictions with those obtained from CT scans. The femurs were meshed with a target element edge length of 3 mm for 10-node tetrahedral elements by using commercially available software (Ansa 22.0.1; Beta CAE Systems, Root, Switzerland).^23^ Materials were mapped to the finite elements representing bone tissue as described previously by Enns-Bray and colleagues.^24^ In the absence of standing scans, hip width and trochanteric soft tissue thickness (TSTT) were estimated from whole-body DXA scans using sex- and ethnicity-specific correction equations to account for overestimation from supine measurements.^11^ The DXA-based biofidelic FEM (Figure 1A) was rotated to represent different fall angles and made to impact at different impact velocities. The biofidelic FEMs were processed in an explicit commercial solver (LS-Dyna, R12.2.1, ANSYS, Inc., Canonsburg, PA, USA). A simulation outcome was classified as a fracture if a 10-mm long area (fracture length) on the surface of the femur exceeded strain thresholds 2 ms after the peak force.^11^ For each simulation, the peak impact force (the contact force between the soft tissue and the floor), peak femur force (the contact force between the hip joint cartilage and the femur head), and fracture outcomes, including fracture lengths, were recorded.

### Biofidelic FEM with hip protector

This study utilized the generic hip protector model developed in a previous study.^20^ Briefly, dimensions were determined using the median values for length, width, and thickness from eleven soft-shell hip protectors, resulting in an elliptically shaped pad measuring 170.0 mm in length, 150.0 mm in width, and 14.5 mm in thickness. A material model for highly compressible low-density foams was utilized (MAT_057) to represent the mechanical properties of the hip protector, characterized by a tensile modulus of 0.002 GPa, a density of 1.00E-7 kg/mm^3^, a hysteresis parameter of 0.05, and a shape parameter of 5.^20^ The positioning and morphing of this generic hip protector onto the DXA-based biofidelic FEM were performed using an automated Python (3.12.3)-based pipeline utilizing open-source packages and commercial software (Ansa 22.0.1; Beta CAE Systems, Root, Switzerland). The pipeline first positions the hip protector over the greater trochanter in a fall position and then morphs it to match the skin surface (Figure 1B).

### Surrogate model

The critical velocity for a given fall angle refers to the minimum impact speed at which the fracture length reaches 10 mm. Various fall configurations are required to be simulated to determine the critical velocity at different fall angles. However, simulating a biofidelic FEM is computationally demanding, with each simulation on 4 cores requiring approximately 24-36 h of real-time processing. As a result, developing a subject-specific surrogate model to minimize the number of required actual simulations is essential. A Gaussian process regression (GPR)-based surrogate model was employed to minimize the number of simulations needed to construct the critical velocity curve. The initial GPR model was built using at least 3 simulations, with the kernel defined as $\mathrm{kernel}=C \cdot \mathrm{RBF} \cdot \mathrm{DotProduct}+\mathrm{WhiteKernel}$. This composite kernel combines a constant kernel $C$to scale the overall variance, a radial basis function (RBF) kernel to capture smooth nonlinear relationships between the inputs (impact velocity and fall angle) and the predicted fracture length, a dot product kernel to represent potential linear trends, and a white noise kernel to account for simulation noise. This kernel combination was selected to flexibly represent both linear and nonlinear dependencies while maintaining robustness with a limited number of training simulations. The same kernel structure was used for all subjects, while the hyperparameters were optimized using the simulation data generated for each subject, resulting in subject-specific surrogate models.

**Figure 2:**
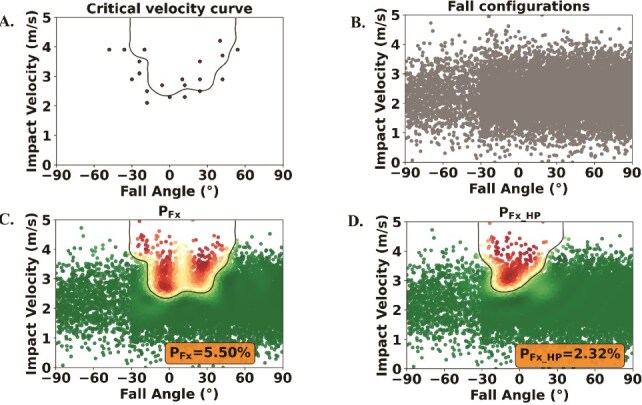
(A) Critical velocity curve based on simulation points. (B) Fall configurations derived from.^27^^,^^28^ (C) P_Fx_ calculation by overlaying critical velocity curve (A) on to fall configurations (C). (D) P_Fx_HP_ calculation by overlaying critical velocity curve from falls with hip protectors on to fall configurations (C). Red points: fall configurations with fractured outcome; green points: fall configurations with non-fractured outcome; P_Fx_: probability of hip fracture; P_Fx_HP_: probability of hip fracture with hip protector.

To enhance prediction accuracy for the critical velocity curve, subsequent sampling points were selected by identifying the points on the critical velocity curve with the highest uncertainty. This process aimed to predict a fracture length of 10 mm involving by optimizing the objective function $f=-{e}^{-1e3{\left({FxL}_{\mathrm{thres}}-{FxL}_{\mathrm{GPR}}\left(\mathrm{IV},\mathrm{FA}\right)\right)}^2} \cdot \sigma$, where $f$ is the objective value to be minimized, ${FxL}_{\mathrm{thres}}$ is the target fracture length, ${FxL}_{\mathrm{GPR}}$ is the GPR predicted fracture length as a function of the impact velocity and fall angle, and $\sigma$ is the GPR predicted uncertainty. Minimizing $f$ corresponds to identifying the impact velocity and angle that maximize uncertainty while predicting the desired fracture length. The GPR model was implemented in Python (3.12.3) using the *Scikit-learn* library (1.4.2),^25^ while the optimization relied on the *SciPy* (1.11.4) minimize function.^26^ This process was repeated until 20 simulations had been completed for a subject. Ultimately, the critical velocity curve was derived from the trained GPR model using the limited simulations (Figure 2A). This procedure was repeated for the biofidelic FEM with a hip protector to obtain another critical velocity curve.

### Computing probability of hip fracture (P_Fx_)

A fall angle probability distribution was derived from reported fall directions based on video data analysis.^27^ Anterior falls, represented from −90° to −30° in this study, made up 12%, sideways falls accounted for 39.36% (ranging from −30° to 30°), and posterior falls represented 48.64% (ranging from 30° to 90°). This probability distribution of fall angles formed the *x*-axis of the fall configuration (Figure 2B). Due to the lack of impact speed data from an Asian cohort, impact speed was assumed to be normally distributed with an average impact speed of 2.14 m/s and a SD of 0.63 m/s, which were derived from video analysis of both protected and unprotected falls in elderly Caucasians.^28^ This normally distributed impact speed formed the *y*-axis of the fall configuration (Figure 2B). Further, evidence from a video analysis has reported that only 40% of real-life falls result in hip impact.^29^ Therefore, the P_Fx_ estimated from our analysis was scaled by a factor of 0.4 to reflect a realistic number of falls that might lead to an impact on the hip. This adjustment enabled the calculation of the probability of hip fracture (P_Fx_; Figure 2C) and the probability of hip fracture with a hip protector (P_Fx_HP_; Figure 2D).

### Statistical analysis

The RR in the hip protector arm of these in-silico trials, assuming full compliance, was calculated by dividing P_Fx_HP_ with P_Fx_ for each subject. Only subjects with a non-zero baseline fracture probability were included in the RR calculation, since RR is undefined when the reference risk is zero. Demographic variables, such as age, weight, height, BMI, TSTT, FRAX-HFP, along with aBMD, and T-score, were compared across the three ethnic groups, stratified by sex. Descriptive statistics, including means and SDs, were computed for both these variables and derived variables (P_Fx_, P_Fx_HP_, and RR). ANOVA was performed to compare the means of continuous variables across the three ethnic groups. Further, Tukey’s honestly significant difference (HSD) test was performed to make pairwise comparisons between group means. Derived variables, along with aBMD, and T-score were adjusted for age. Subjects with FRAX-HFP more than the threshold of 2% were classified as high risk as per FRAX-HFP.^30^ Subjects with P_Fx_ greater than the highest tertile were categorized as being at high risk of hip fracture, while all others were considered low risk. The highest tertile approach was chosen because there is no established threshold, and it minimizes sensitivity to outliers. Agreement between FRAX-HFP and P_Fx_ classifications was assessed using Cohen’s kappa, which quantifies agreement beyond chance.^31^ Systematic differences in classification were evaluated using McNemar’s test on paired binary classifications, testing whether the proportion of subjects classified differently by the two tools was statistically significant. Additionally, reclassification metrics were calculated to quantify the clinical impact of P_Fx_ relative to FRAX-HFP. The proportion of FRAX-HFP high-risk subjects reclassified as low-risk by P_Fx_ was determined as the down-classification percentage. To further understand the net clinical impact, the net down-classification metric was calculated by subtracting the up-classifications (subjects reclassified from FRAX-HFP low-risk to P_Fx_ high-risk) from the down-classifications (subjects reclassified from FRAX-HFP high-risk to P_Fx_ low-risk), with the result normalized by the total number of subjects. Subjects with RR values exceeding 0.4, as reported in a systematic review of hip protector randomized controlled trials (RCTs)^16^ were assumed to have high RR and vice versa. Odds ratios (ORs) and corresponding 95% CIs were calculated using 2 × 2 contingency tables to evaluate the risk of hip fracture across ethnicities. The *p*-values were derived from Fisher’s exact test, implemented using the “*statsmodels*” Python package (version 0.14.4). The Mann-Whitney U test was applied for non-parametric comparisons between the following groups: (1) mean P_Fx_ and P_Fx_HP_ in the overall sample and by ethnicity and sex; (2) mean baseline variables between subjects with zero and non-zero P_Fx_ in the pooled sample; (3) high and low fracture risk groups; and (4) high and low RR groups All statistical tests were conducted using the “*scipy*” Python package (v1.13.1), with a significance threshold of *p* < .05. Scatter plots of P_Fx_ and RR relative to T-score, FRAX-HFP, and TSTT were generated to examine the relationships between these variables. These distributions were assessed in relation to different thresholds: the osteoporotic T-score threshold of −2.5 and the osteopenic threshold of −1.

## Results

The study included a total of 700 subjects, including males and females (females: 375; mean age: 71.53 yr [SD: 7.46]), as detailed in Table 1. Significant differences between males and females were noted in age (*p* = .020), weight (*p* < .001), height (*p* < .001), TSTT (*p* < .001) and FRAX-HFP (*p* = .015) though BMI did not differ significantly (*p* = .189). After adjusting for age, aBMD was significantly different between males and females (*p* < .001), however, T-score did not differ significantly (*p* = .87). The overall P_Fx_ was 4.49% (SD: 4.71%), with males showing a mean P_Fx_ of 5.56% (SD: 5.17%) and females showing a mean P_Fx_ of 3.56% (SD: 4.05%) (*p* < .001). Ethnic differences were significant across all measured variables except age, for both males and females (*p* < .01). Post hoc comparisons showed that Chinese subjects, regardless of sex, had significantly higher FRAX-HFP and P_Fx_ values than both Indian and Malay subjects (*p* ≤ .01). No significant differences were found between the Indian and Malay groups (*p* > .05).

**Table 1:** Subject characteristics included in the study.

**Variable**	**Pooled**	**Chinese**	**Indian**	**Malay**	***p*-value**			
					**Overall**	**Chinese vs Indian**	**Chinese vs Malay**	**Indian vs Malay**
**Males**	325	140	69	116				
**Age (yr)**	72.17 (7.43)	72.62 (7.12)	71.19 (8.17)	72.22 (7.34)	.423	.390	.901	.635
**Weight (kg)**	68.29 (12.12)	65.43 (9.41)	71.71 (13.91)	69.71 (13.18)	**.001**	**.001**	**.012**	.511
**Height (cm)**	165.05 (5.75)	164.83 (5.01)	167.15 (6.73)	164.07 (5.68)	**.002**	**.015**	.534	**.001**
**BMI (kg/m** ^**2**^ **)**	25.02 (3.93)	24.06 (3.11)	25.56 (4.11)	25.85 (4.45)	**.001**	**.024**	**.001**	.871
**TSTT (cm)**	2.77 (0.70)	2.43 (0.55)	3.16 (0.75)	2.94 (0.65)	**<.001**	**<.001**	**<.001**	.052
**FRAX-HFP (%)**	3.41 (2.94)	4.54 (3.31)	2.11 (2.15)	2.82 (2.33)	**<.001**	**<.001**	**<.001**	.208
**aBMD (g/cm** ^**2**^ **)**	0.76 (0.14)	0.72 (0.13)	0.84 (0.16)	0.76 (0.13)	**<.001**	**<.001**	**.049**	**<.001**
**aBMD (g/cm** ^**2**^ **)** ^**a**^	0.76 (0.14)	0.73 (0.13)	0.84 (0.15)	0.77 (0.12)	**<.001**	**<.001**	.053	**.001**
**T-score**	−1.65 (1.12)	−1.96 (1.01)	−1.04 (1.26)	−1.64 (1.01)	**<.001**	**<.001**	.052	**<.001**
**T-score** ^**a**^	−1.62 (1.08)	−1.91 (1.00)	−1.05 (1.16)	−1.61 (0.99)	**<.001**	**<.001**	.061	**.001**
**P**_**Fx**_ **(%)**	5.69 (5.36)	7.68 (5.37)	3.30 (4.70)	4.70 (4.88)	**<.001**	**<.001**	**<.001**	.168
**P**_**Fx**_ **(%)**^**a**^	5.56 (5.17)	7.47 (5.27)	3.37 (4.19)	4.56 (4.81)	**<.001**	**<.001**	**<.001**	.248
**Females**	375	169	80	126				
**Age (yr)**	70.98 (7.46)^b^	71.54 (7.01)	71.81 (8.39)	69.71 (7.30)^b^	.060	.960	.092	.117
**Weight (kg)**	59.51 (11.46)^b^	55.58 (9.48)^b^	62.93 (10.73)^b^	62.61 (12.70)^b^	**<.001**	**<.001**	**<.001**	.977
**Height (cm)**	152.70 (6.12)^b^	154.13 (6.13)^b^	153.79 (5.92)^b^	150.09 (5.38)^b^	**<.001**	.901	**<.001**	**<.001**
**BMI (kg/m** ^**2**^ **)**	25.53 (4.76)	23.39 (3.80)	26.61 (4.29)	27.70 (5.01)^b^	**<.001**	**<.001**	**<.001**	.185
**TSTT (cm)**	3.69 (1.19)^b^	3.06 (0.96)^b^	4.23 (0.99)^b^	4.21 (1.17)^b^	**<.001**	**<.001**	**<.001**	.984
**FRAX-HFP (%)**	3.23 (3.78)^b^	4.36 (4.22)	1.81 (1.81)	2.61 (3.64)	**<.001**	**<.001**	**<.001**	.278
**aBMD (g/cm** ^**2**^ **)**	0.64 (0.12)	0.62 (0.10)	0.69 (0.13)	0.64 (0.11)	**<.001**	**<.001**	.143	**.017**
**aBMD (g/cm** ^**2**^ **)** ^**a**^	0.64 (0.11)^b^	0.62 (0.10)^b^	0.69 (0.12)^b^	0.64 (0.11)^b^	**<.001**	**<.001**	.366	**.001**
**T-score**	−1.61 (1.07)	−1.82 (0.94)	−1.15 (1.21)	−1.61 (1.07)	**<.001**	**<.001**	.199	**.007**
**T-score** ^**a**^	−1.63 (1.01)	−1.82 (0.88)	−1.14 (1.13)	−1.69 (0.99)	**<.001**	**<.001**	.503	**<.001**
**P**_**Fx**_ **(%)**	3.46 (4.28)	4.57 (4.68)	2.07 (3.14)	2.84 (3.98)	**<.001**	**<.001**	**.001**	.392
**P**_**Fx**_ **(%)**^**a**^	3.56 (4.05)^b^	4.57 (4.57)^b^	2.01 (2.82)	3.20 (3.61)	**<.001**	**<.001**	**.010**	.087

Abbreviations: aBMD, areal BMD; FRAX-HFP, fracture risk assessment tool-hip fracture probability; P_Fx_, probability of hip fracture; TSTT, trochanteric soft tissue thickness.

^a^Adjusted for age.

^b^Significance observed between males and females.

Bold values indicate statistically significant differences (p < 0.05). Values are mean (SD) for continuous variables; overall *p*-value: difference in means between different ethnicities; *p*-value: difference between each individual pair of ethnicities.

Using the top tertile P_Fx_ value (>5%) as a threshold for high risk, 152 males (47%; 91 Chinese, 17 Indians, 44 Malays) and 102 females (27%; 63 Chinese, 12 Indians, 27 Malays) were classified as high risk. These subjects were significantly older and had lower weight, BMI, T-score, and TSTT, as well as higher FRAX-HFP scores (*p* < .001) than their low-risk counterparts. Among subjects with no hip fracture risk at baseline (P_Fx_ = 0%; n = 66; 13 Chinese, 26 Indian, 27 Malay), pooled analysis showed they were significantly younger, had greater weight and BMI, higher T-scores, and greater TSTT compared to those with a non-zero P_Fx_ (P_Fx_ > 0%, n = 634, *p* < .001).

The agreement between FRAX-HFP and P_Fx_ classifications was limited in both the overall cohort and the osteopenic subgroup. In the overall cohort, Cohen’s kappa indicated weak agreement (κ = 0.48). A total of 227 subjects were classified as high-risk and 284 as low-risk by both tools. In contrast, 162 subjects classified as high-risk by FRAX-HFP were down-classified to low-risk by P_Fx_, while 27 subjects classified as low-risk by FRAX-HFP were up-classified to high-risk by P_Fx_. McNemar’s test confirmed a significant difference between the classifications (*p* < .001). Among those classified as high-risk by FRAX-HFP, 41.65% were reclassified as low-risk by P_Fx_, resulting in a net down-classification of 19.30%. In the osteopenic subgroup, agreement was even lower, with Cohen’s kappa indicating minimal agreement (κ = 0.27). Of the 230 subjects classified as high-risk by FRAX-HFP, 126 (54.78%) were down-classified by P_Fx_, and 22 subjects were up-classified. McNemar’s test again showed significant differences between the tools (*p* < .001), with a net down-classification of 27.40%.

ORs for hip fracture risk by ethnicity are provided in Table 2. After adjusting for age, Chinese males faced up to 4.5 times higher risk of hip fracture than Indians and 3.15 times higher than Malays (both *p* < .001), while Chinese females had up to 7.7 times higher risk than Indians (*p* = .001) and 2.22 times higher than Malays (*p* = .016). No significant differences in ORs were found between Indians and Malays in either sex.

**Table 2:** The association between ethnicity and hip fracture risk.

**Comparison**	**Odds ratio (95% CI)**	***p*-value**	**Age-adjusted odds ratio (95% CI)**	***p*-value**
**Chinese vs Indian (Males)**	5.26 (2.49–11.12)	**<.001**	4.57 (2.21–9.44)	**<.001**
**Chinese vs Malay (Males)**	3.25 (1.87–5.64)	**<.001**	3.15 (1.81–5.49)	**<.001**
**Indian vs Malay (Males)**	0.62 (0.28–1.38)	.239	0.69 (0.32–1.51)	.353
**Chinese vs Indian (Females)**	3.13 (1.33–7.35)	**.009**	7.70 (2.30–25.76)	**.001**
**Chinese vs Malay (Females)**	2.40 (1.24–4.65)	**.009**	2.22 (1.16–4.24)	**.016**
**Indian vs Malay (Females)**	0.77 (0.30–1.99)	.586	0.29 (0.08–1.03)	.056

Bold values indicate statistically significant differences (p < 0.05).

With regard to the effectiveness of hip protectors, a total of 210 subjects (females:105; mean age: 72.45 yr (SD: 7.85)) were evaluated, as detailed in Table 3. The mean P_Fx_HP_ dropped to 3.50% (SD: 4.14%) in males from a mean P_Fx_ of 5.56% (SD: 5.17%) and to 2.91% (SD: 3.59%) from a mean P_Fx_ of 3.56% (SD: 4.05%) in females, showing a significant difference between without and with hip protectors (*p* < .001). In males, P_Fx_HP_ was significantly lower than P_Fx_ within each ethnic subgroup (Chinese (*p* < .001), Indians (*p* = .023), Malay (*p* = .003)). Among females, P_Fx_HP_ was significantly reduced within the Chinese (*p* < .001), and Indian (*p* = .041); however, there was a non-significant reduction for Malay (*p* = .092). Among those whose risk reduced to zero after wearing hip protectors (P_Fx_HP_ of 0% (N = 24; 3 Chinese, 14 Indians, 7 Malays), pooled analysis showed that they had significantly higher T-scores (*p* < .001), and greater TSTT (*p* = .001) compared to those with a non-zero P_Fx_HP_ (N = 172).

**Table 3:** Efficacy of hip protectors (*N* = 210).

**Variable**	**Pooled**	**Chinese**	**Indian**	**Malay**	***p*-value**			
					**Overall**	**Chinese vs Indian**	**Chinese vs Malay**	**Indian vs Malay**
**Males**	105	35	35	35				
**Age (yr)**	73.44 (7.38)	73.51 (5.74)	73.80 (8.76)	73.00 (7.53)	.902	.986	.955	.895
**Weight (kg)**	64.44 (9.98)	63.45 (10.26)	66.32 (10.79)	63.54 (8.80)	.397	.456	.999	.477
**Height (cm)**	166.03 (5.74)	166.73 (4.55)	166.52 (7.10)	164.84 (5.25)	.323	.988	.357	.439
**BMI (kg/m** ^**2**^**)**	23.33 (3.14)	22.83 (3.63)	23.83 (3.02)	23.35 (2.70)	.413	.379	.769	.796
**TSTT (cm)**	2.58 (0.56)	2.25 (0.52)	2.88 (0.47)	2.61 (0.51)	**<.001**	**<.001**	**.009**	.075
**FRAX-HFP (%)**	3.76 (3.40)	5.51 (4.31)	2.55 (2.32)	3.24 (2.53)	**<.001**	**.001**	**.010**	.640
**aBMD (g/cm** ^**2**^ **)**	0.75 (0.15)	0.70 (0.11)	0.80 (0.16)	0.74 (0.15)	**.015**	**.011**	.504	.170
**aBMD (g/cm** ^**2**^ **)** ^**a**^	0.75 (0.14)	0.71 (0.12)	0.81 (0.15)	0.74 (0.15)	**.009**	**.007**	.517	.117
**T-score**	−1.76 (1.18)	−2.13 (0.90)	−1.33 (1.30)	−1.81 (1.19)	**.015**	**.012**	.486	.182
**T-score** ^**a**^	−1.71 (1.14)	−2.08 (0.99)	−1.26 (1.13)	−1.79 (1.15)	**.009**	**.007**	.512	.117
**P**_**Fx**_ **(%)**	6.79 (5.54)	8.57 (5.12)	5.03 (5.72)	6.76 (5.35)	**.027**	**.020**	.345	.377
**P**_**Fx**_ **(%)**^**a**^	6.56 (5.19)	8.32 (5.07)	4.72 (4.94)	6.63 (5.05)	**.013**	**.009**	.341	.252
**P**_**Fx_HP**_ **(%)**	3.70 (4.52)	4.63 (4.83)	3.00 (4.41)	3.48 (4.29)	.302	.289	.534	.898
**P**_**Fx_HP**_ **(%)**^**a**^	3.50 (4.14)	4.41 (4.67)	2.72 (3.81)	3.36 (3.83)	.228	.204	.539	.791
**RR**	0.38 (0.30)	0.42 (0.29)	0.34 (0.32)	0.38 (0.28)	.546	.514	.845	.844
**RR** ^**a**^	0.38 (0.28)	0.42 (0.29)	0.33 (0.29)	0.38 (0.26)	.458	.426	.854	.752
**Females**	105	35	35	35				
**Age (yr)**	71.46 (8.21)^b^	69.94 (7.69)^b^	72.31 (8.20)	72.11 (8.74)	.411	.452	.513	.994
**Weight (kg)**	56.18 (10.63)^b^	51.79 (10.06)^b^	60.56 (9.50)^b^	56.18 (10.72)^b^	**.002**	**.001**	.170	.170
**Height (cm)**	153.26 (7.11)^b^	156.00 (5.42)^b^	154.29 (6.56)^b^	149.49 (7.67)^b^	**<.001**	.527	**<.001**	**.009**
**BMI (kg/m** ^**2**^ **)**	23.88 (4.01)	21.26 (3.83)	25.43 (3.56)^b^	24.95 (3.34)	**<.001**	**<.001**	**<.001**	.843
**TSTT (cm)**	3.51 (1.16)^b^	2.63 (0.91)	4.17 (0.89)^b^	3.73 (1.09)^b^	**<.001**	**<.001**	**<.001**	.144
**FRAX-HFP (%)**	2.90 (2.75)^b^	4.10 (3.53)	2.03 (1.75)	2.56 (2.28)	**.004**	**.004**	**.041**	.679
**aBMD (g/cm** ^**2**^ **)**	0.63 (0.12)	0.60 (0.10)	0.67 (0.13)	0.63 (0.13)	**.031**	**.023**	.457	.304
**aBMD (g/cm** ^**2**^ **)** ^**a**^	0.63 (0.11)^b^	0.59 (0.10)^b^	0.67 (0.12)^b^	0.63 (0.11)^b^	**.005**	**.003**	.215	.227
**T-score**	−1.68 (1.13)	−2.02 (0.93)	−1.31 (1.19)	−1.70 (1.17)	**.031**	**.023**	.454	.308
**T-score** ^**a**^	−1.73 (1.05)	−2.14 (0.88)	−1.32 (1.09)	−1.72 (1.03)	**.004**	**.003**	.190	.225
**P**_**Fx**_ **(%)**	5.06 (5.05)	7.46 (4.70)	2.66 (3.53)	5.05 (5.62)	**<.001**	**<.001**	.085	.090
**P**_**Fx**_ **(%)**^**a**^	5.28 (4.85)	8.03 (4.82)	2.69 (3.08)	5.12 (4.94)	**<.001**	**<.001**	**.017**	.056
**P**_**Fx_HP**_ **(%)**	2.71 (3.82)	3.33 (3.88)	1.46 (2.50)	3.34 (4.57)	.059	.098	1.000	.096
**P**_**Fx_HP**_ **(%)**^**a**^	2.91 (3.59)	3.85 (3.61)	1.49 (2.46)	3.41 (4.12)	**.012**	**.015**	.856	.058
**RR**	0.42 (0.28)	0.38 (0.23)	0.38 (0.31)	0.51 (0.29)	.122	.998	.170	.175
**RR** ^**a**^	0.43 (0.28)	0.41 (0.21)	0.37 (0.32)	0.51 (0.29)	.147	.870	.313	.146

Abbreviations: aBMD, areal BMD; FRAX-HFP, fracture risk assessment tool-hip fracture probability; P_Fx_, probability of hip fracture; P_Fx_HP_, P_Fx_ with hip protectors; RR, relative risk; TSTT, trochanteric soft tissue thickness.

^a^Adjusted for age.

^b^Significance observed between males and females.

Bold values indicate statistically significant differences (p < 0.05). Values are mean (SD) for continuous variables; overall *p*-value: difference in means between different ethnicities; *p*-value: difference between each individual pair of ethnicities.

The overall RR of hip fracture with a fully compliant hip protector use was 0.40 (SD: 0.28), with a 62% risk reduction in males (RR: 0.38, SD: 0.28) and a 57% reduction in females (RR: 0.43, SD: 0.28), with no significant sex-based difference (*p* = .406). No significant ethnic differences in RR were observed within either sex (*p* = .458 for males; *p* = .147 for females). A total of 45 males (19 Chinese, 13 Indians, 13 Malays) and 45 females (17 Chinese, 10 Indians, 18 Malays), each representing 43% of their respective groups, were classified as having a high RR. Within this high-RR subgroup, both males and females had significantly lower T-scores (*p* < .001).

**Figure 3:**
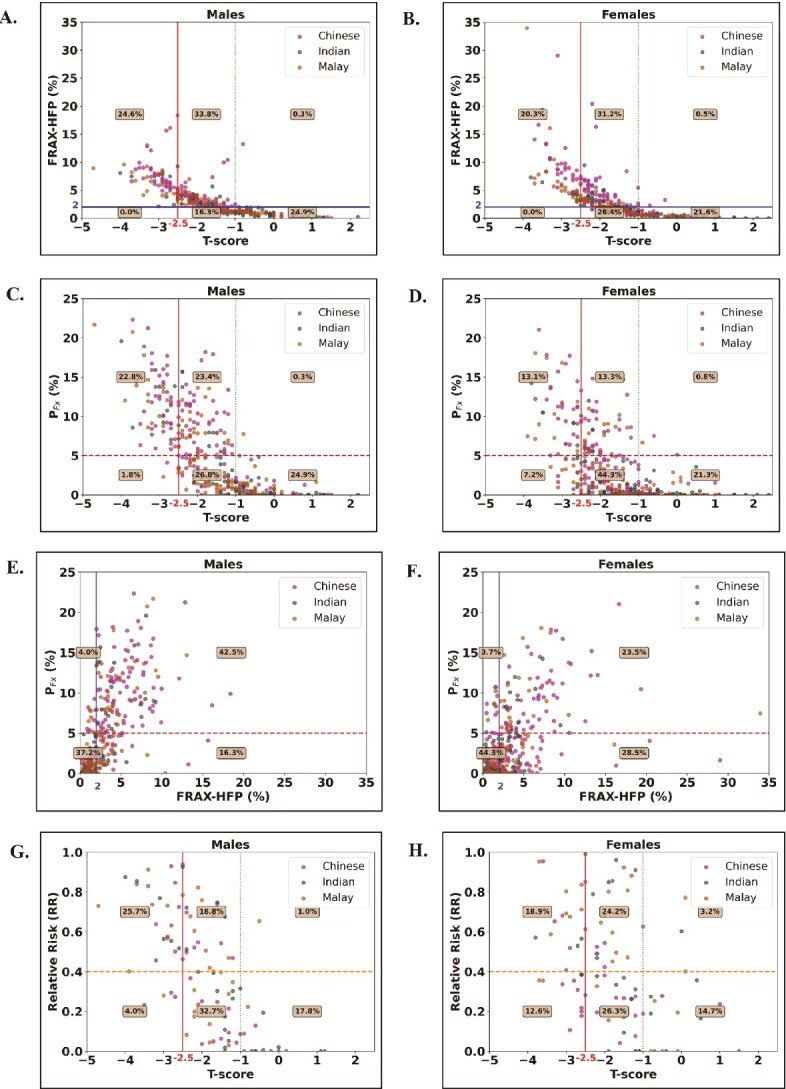
(A, B) FRAX-HFP with respect to T-score; P_Fx_ with respect to (C, D) T-score; (E, F) FRAX-HFP; (G, H) relative risk for the usage of hip protectors with respect to T-score (*N* = 196). Solid red line (vertical): osteoporotic T-score threshold (T-score = −2.5); dashed green line (vertical): normal T-score threshold (T-score = −1); dashed brown line (horizontal): P_Fx_ threshold at 5%: highest tertile value of pooled P_Fx_ in this study; solid blue line (vertical): FRAX-HFP threshold at 2%^30^; dashed orange line (horizontal): RR threshold of 0.4.^16^ FRAX-HFP: fracture risk assessment tool-hip fracture probability; P_Fx_: probability of hip fracture.

The distributions of FRAX-HFP, P_Fx_, and RR in relation to T-scores, as well as P_Fx_ in relation to FRAX-HFP are shown in Figure 3. Using the FRAX-HFP threshold of 2%,^30^ 59% of males and 52% of females were identified as at risk, whereas P_Fx_ identified only 47% of males and 27% of females at risk of hip fracture. Among osteoporotic subjects, FRAX flagged 100% for both males and females, while P_Fx_ identified 93% of males and 64% of females as high risk. For osteopenic subjects, FRAX marked 69% of males and 55% of females as at-risk, compared to 47% of males and 23% of females by P_Fx_, with just a 40% overlap for males and 18% for females between the two methods. As for RR, 55% of males and 49% of females with low bone mass had a high RR, indicating limited effectiveness of hip protectors in reducing fracture risk for this group. Additionally, 96% of males and 86% of females with normal bone mass exhibited a low RR or high-risk reduction.

## Discussion

The primary aim of this study was to predict the P_Fx_ using biofidelic FEMs built from clinical DXA images of a cohort of community-dwelling older adults. Our results for P_Fx_ highlight the complex etiology of hip fractures, which involves both reduced bone strength and increased mechanical loading during impact.^1^ Additionally, the inverse relationship between fracture risk and TSTT supports the importance of biomechanical models that incorporate the full pelvic structure, including soft tissue components.^19^ Previous research on hip fracture trends in Singapore^32^ reported the highest incidence among Chinese females (264 per 100 000), followed by Malays (185 per 100 000) and Indians (141 per 100 000). Our analysis follows similar trends, although we demonstrate higher odds of hip fracture risk in Chinese females than both Malay (OR: 2.22) and Indian females (OR: 7.70) than reflected by the population data. Similar trends were demonstrated in males, with Chinese males exhibiting higher odds than their Malay (OR: 3.15) and Indian (OR: 4.57) counterparts. These results suggest that DXA-based biofidelic FEMs reliably reflect population-specific hip fracture trends.

Our analysis shows that P_Fx_ has a relatively weak agreement with FRAX-HFP across the overall cohort but consistently classifies fewer subjects as high-risk. In the osteopenic subgroup, agreement is minimal, with more than half of FRAX-HFP high-risk subjects being down-classified by P_Fx_. These findings highlight a discordance between the two approaches, which may partly arise because P_Fx_, derived from DXA-based biofidelic FEMs, provides biomechanical insights not captured by traditional clinical assessments, particularly in osteopenic subjects.^7^ Clinically, this selectivity could help reduce the risk of overtreatment by restricting high-risk classifications to subjects with the highest predicted fracture probability, supporting more personalized intervention strategies. These findings highlight the potential value of P_Fx_ as a complementary tool to current clinical standards, improving risk stratification and guiding more targeted prevention efforts in populations with diverse fracture risk profiles. The study also found that Chinese populations exhibited higher biomechanical (P_Fx_) and clinical (FRAX-HFP) risks, indicating a greater susceptibility to fractures. In contrast, lower P_Fx_ and FRAX-HFP scores in Indian and Malay populations suggest a reduced fracture risk, though subject-specific assessments remain critical.

Our analysis also showed that, on average, males had a higher P_Fx_ than females within the cohort, which contrasts with the commonly observed higher fracture incidence in females in real-world data. Additionally, while T-scores were nearly identical between males and females, FRAX-HFP was significantly higher in males within this cohort. The higher P_Fx_ observed in males suggests a greater biomechanical risk of fracture upon falling, consistent with these clinical indicators. Although females experience a higher overall incidence of hip fractures,^33^ epidemiological studies have also shown that females tend to experience falls more frequently than males.^34^ In community-dwelling older adults in Singapore, reported fall incidence rates were 31.1% in females compared with 12.5% in males.^35^ Because P_Fx_ in our model represents the probability of sustaining a fracture given a fall, it does not incorporate sex-specific differences in fall frequency. Consequently, the higher P_Fx_ observed in males in our simulations reflects modeled biomechanical susceptibility per fall within this cohort. In contrast, the higher fall frequency reported among females in epidemiological studies may substantially contribute to the greater overall hip fracture incidence observed in females at the population level. This distinction highlights an important limitation shared with other bone health markers, including DXA-based T-score and FRAX HFP, in that they are interpreted independently of fall exposure, and emphasizes the complementary roles of biomechanical susceptibility and fall risk in determining fracture incidence. Our research highlights the need for sex- and ethnicity-specific approaches and suggests that integrating both P_Fx_ and FRAX-HFP into clinical decision-making could improve fracture risk stratification.

A secondary aim was to assess the RR assuming full compliance of hip protectors within this cohort. Our analysis demonstrated that hip protectors significantly reduce both P_Fx_HP_ and RR. The P_Fx_ was reduced by 62% in males and 57% in females, highlighting their effectiveness in preventing hip fractures in both sexes. Although the reduction in P_Fx_ for Malay females was not statistically significant, this was likely due to the smaller sample size in this subgroup. Our models showed that P_Fx_HP_ reduced to zero for 11.4% of subjects (N = 24/210) indicating the effectiveness of hip protectors. The pooled RR observed in this study aligns with findings from a systematic review of 4 RCTs, which reported a mean RR of 0.40 for hip protectors RR.^16^ Assuming partial compliance to better represent real-world use, we incorporated a 50% compliance rate.^37^^,^^38^ This resulted in adjusted RRs of 0.69 for males and 0.72 for females, which fall within the published range at this level of compliance (RR: 0.67-0.82).^15^^,^^36^ Our results also demonstrated that RR is highly subject-specific, specifically those with lower T-scores exhibiting higher RRs, meaning lower risk reductions. Moreover, the average RR of 0.40 observed in our study is more favorable than the average RR of 0.68 for pharmacological interventions, as reported in a systematic review of 33 RCTs.^39^ This suggests that hip protectors may be less effective for osteoporotic subjects and combining them with other preventive measures could be beneficial in preventing fractures.

There are several limitations associated with our study. The first limitation is the absence of data on actual fractures, which could have further validated the modeling technology used to assess hip fracture risk. Second, our study used tertile-based stratification of P_Fx_ for the studied sample. In the absence of longitudinal fracture outcome data, we are unable to assess how this threshold corresponds to observed fracture incidence. Therefore, its clinical relevance and generalizability remain uncertain and require validation in independent cohorts with prospective fracture outcomes. Further, this modeling framework focuses on the biomechanical consequences of a fall event once it occurs and does not account for physiological or environmental factors that influence fall occurrence. Additionally, the fall angle distribution^27^ and impact speed distribution^28^ employed in our analysis were based on video observations of falls in Caucasian populations, which may not be directly applicable to Asian populations. Although these represent the best available empirical data, future studies should aim to characterize these parameters across diverse ethnic groups, especially considering that the majority of global hip fractures are projected to occur in Asia by 2050.^40^

In conclusion, we have developed a DXA-based, in-silico fracture risk assessment tool that incorporates fall-related factors to estimate personalized hip fracture risk (P_Fx_). This tool enables the execution of in-silico randomized controlled trials at a population level, offering quantitative insights into the effectiveness of interventions such as hip protectors. Our findings align with observed trends in hip fractures among older adults in Singapore, emphasizing the roles of sex and ethnicity in fracture risk. Additionally, we demonstrated that consistent use of hip protectors significantly reduces P_Fx_, highlighting the potential of this intervention. While the FRAX tool captures long-term clinical risk on a population basis, P_Fx_ offers a more immediate, individualized assessment of biomechanical vulnerability during a fall. The two methods identified different high-risk groups, with FRAX-HFP highlighting a broader at-risk population. This suggests that P_Fx_ may refine risk stratification, particularly in osteopenic individuals, where treatment decisions remain uncertain. Together, these results highlight the clinical value of our DXA-based tool in optimizing fracture prevention efforts, such as the targeted use of hip protectors.

## Data Availability

The data underlying this article cannot be shared publicly due to restrictions in place at the time of cohort data collection and to protect participant privacy.
